# Practical Notes and Formulæ

**Published:** 1884-05-20

**Authors:** 


### PRACTICAL NOTES AND FORMULÆ.

**Carbolic Acid Injections in Hæmorrhoids.**—Dr. J. W. Girrard, of Winchester, Tenn., asks, in the March number of the Therapeutic Gazette, for information in regard to the carbolic acid treatment of piles. I would say that I have used it in several cases with favorable results and no accident. Here is the formula that I use:

℞ Acidi carbolici, }

Napthalini, } ........................aa ʒ j.

Morphinæ acetatis, }

Hydrast. sulphatis, } ........................aa gr. j.

Aquæ dest., ........................ʒ iij.

Mix. ft sol.

Sig. Inject into tumor through the base every four days till cured.

As a local anæsthetic:—

℞ Etheris sulphurici........................℥ ij.

Camphoræ.... ........................℈ ij.

M.

As an ointment:—

℞ Acidi tannici, }

Hydrarg.' chlorid. mitis, } ........................aa ʒ j.

Ol. lini, }

Ol. origani, } ........................aa ʒ ij.

M. Sig. Apply externally.

Let the doctor try this and he need not be afraid of any dangerous results.

—*Therapeutic Gazette.*

**Glycerine in Febrile Conditions.**—M. Semmola has found glycerine of great utility in fevers of long duration, like typhoid fever, in arresting denutrition, and limiting febrile consumption. It is particularly indicated when it is feared that the administration of alcohol, so common to-day, may have too exciting an action on the nerve-centres.

M. Semmola recommends the following mixture:

℞ Glycerin. pur., ........................℥ j.

Ac. citric., ........................gr. xxx.

Aq. destil., ........................℥ xvj.

M. One or two tablespoonfuls every hour.—*Med. and Surg. Rep.*

**Scarlatina.**—In a child, say three or four years old, we would take :

℞ Tr. aconit. rad.,............................gtt. ij.
Tr. belledonna............................gtt. iij.
Aquæ....................................℥ jv.

M. Sig. One teaspoonful every hour. A warm bath morning and evening. After each ablution a thorough inunction all over with

Adipis....................................℥ jv.

Quiniæ sulph.............................ʒ j.

M.

—*Amer. Med. Journal.*

**Dr. Mittauer's Aperient Mixture (Alkaline Mixture of Aloes.)**—

℞ Aloes....................................2½ ozs.
Bicarbonate of sodium........................6 "
Comp. spirit lavender........................2 "
Water ......................................4 pints.

M. Maccurate for two weeks and filter. Dose: 1 fl. dr. to 1 fl. oz. half an hour after meals, for costiveness.

—*American Druggist.*

**Emulsion of Copaiba.**—Query. How best to dispense ?

℞ Balsam of copaiba...........................ʒ jv.

Powdered extract of licorice, }

Powdered acacia, } ....................qs.

Camphor water enough to make................℥ iij

*Ans.* Place in a dry mortar 6 drachms each of powdered acacia and extract of licorice (thoroughly dry) and pour on the balsam. Mix well, and add at one time, 12 drachms of camphor water. Continue stirring until the mixture is complete, scraping the sides of the mortar and pestle occasionally. Then add the camphor water little by little, until the total amount of 3 ounces is obtained.

—*American Druggist.*

**Tolu Rock and Rye.**—(Ind. Pharmacy.)

℞ Good whisky..............................1 gallon.
Rock candy ................................4 pounds.
Gum tolu...................................2 ounces.

Put the whole into a two-gallon jug, set in a warm place, and agitate several times a day until the candy is dissolved. Then strain through flannel or coarse muslin.

[The proportion of sugar appears to us larger than good whisky will dissolve, and it may be remarked in passing, that the original "rock and rye" contained figs among its ingredients.—*Ed. Amer. Druggist.*]

**Spermatorrhœa.**—A mixture containing tincture of perchloride of iron and tincture of nux vomica should be given twice or three times a day; also, a pill containing a fourth or a third of a grain of extract of belladonna, with three grains of camphor, should be given at first every night, immediately before going to bed. If these lines of treatment be adhered to, the patient, whether suffering from real spermatorrhœa or simply from frequently returning nocturnal emissions, will steadily improve, and the emissions will occur less and less frequently, till, in the course of a few weeks, or possibly months—for a malady of long standing (as this usually is) is never cured immediately—they will cease altogether, or only occur at such intervals as may be deemed normal, and in which there is no harm whatever.

**Dry Seborrhœa of the Scalp.**—This affection, which is almost always accompanied by some degree of irritation of the scalp, is best treated by the following preparation.

| | | |
|---|---|---|
| ℞ | Zinci. oxid., Sulphur. præcip., } | aa gr. j, |
| | Ung. simpl. | ℥ j. |

M. This ointment should be applied to the head each evening, and carefully washed off in the morning.—*Amer. Med. Digest.*

**A New Chewing Gum.**—

| | | | |
|---|---|---|---|
| ℞ | Venice turpentine | 100 | parts, |
| | American Thus | 75 | " |
| | Yellow wax | 50 | " |
| | Bals. tolu | 10 | " |
| | Bals. Peru | 5 | " |

Melt together and add, in fine powder:

| | | |
|---|---|---|
| Cinnamon (Chinese) | 30 | parts, |
| Chocolate | 50 | " |
| Red Sandal Wood | 10 | " |
| Myrrh | 5 | " |
| Galangal | 5 | " |
| Ginger | 5 | " |
| Cardamom | 2½ | " |

(Parts by weight.) Mix and roll out, when cool enough, into sticks, or make into any suitable form.—*New Idea.*

**Congestive Headache.—Nickel.**—After a number of experiments, Dr. J. M. DaCosta decides that the bromide of nickel is much more effective in the congestive form of headache than any other preparation of bromide. Its dose is from five to seven grains.

**Disguising the Taste of Tincture of Iron.**—Dr. Hager recommends that tincture of the sesqui-chloride of iron be mixed with simple syrup, and then with milk. This mixture will not affect the teeth, nor will the styptic taste be apparent.—*Amer. Drug.*

